# Deforestation Induced Climate Change: Effects of Spatial Scale

**DOI:** 10.1371/journal.pone.0153357

**Published:** 2016-04-21

**Authors:** Patrick Longobardi, Alvaro Montenegro, Hugo Beltrami, Michael Eby

**Affiliations:** 1 Climate & Atmospheric Sciences Institute (CASI), St. Francis Xavier University, Antigonish, NS, Canada; 2 Department of Earth Sciences, St. Francis Xavier University, Antigonish, NS, Canada; 3 Department of Geography, Ohio State University, Columbus, OH, United States of America; 4 School of Earth and Ocean Sciences, University of Victoria, Victoria, BC, Canada; 5 Câmpus do Litoral Paulista Univ Estadual Paulista, São Vicente, SP, Brazil; Universidade de Vigo, SPAIN

## Abstract

Deforestation is associated with increased atmospheric CO_2_ and alterations to the surface energy and mass balances that can lead to local and global climate changes. Previous modelling studies show that the global surface air temperature (SAT) response to deforestation depends on latitude, with most simulations showing that high latitude deforestation results in cooling, low latitude deforestation causes warming and that the mid latitude response is mixed. These earlier conclusions are based on simulated large scal land cover change, with complete removal of trees from whole latitude bands. Using a global climate model we examine the effects of removing fractions of 5% to 100% of forested areas in the high, mid and low latitudes. All high latitude deforestation scenarios reduce mean global SAT, the opposite occurring for low latitude deforestation, although a decrease in SAT is simulated over low latitude deforested areas. Mid latitude SAT response is mixed. In all simulations deforested areas tend to become drier and have lower SAT, although soil temperatures increase over deforested mid and low latitude grid cells. For high latitude deforestation fractions of 45% and above, larger net primary productivity, in conjunction with colder and drier conditions after deforestation cause an increase in soil carbon large enough to produce a net decrease of atmospheric CO_2_. Our results reveal the complex interactions between soil carbon dynamics and other climate subsystems in the energy partition responses to land cover change.

## Introduction

Agricultural lands occupy approximately 38% of the Earth’s land surface [1]. These croplands and pastures presently cover about 10%, 45% and 27% of the areas originally occupied by boreal, temperate, and tropical forests respectively [1–4]. Population growth and the associated expansion of agricultural lands is the primary cause of present day deforestation [4, 5]. Although rates of deforestation have decreased over the last decade, the loss of forested areas is expected to continue during the present century [6, 7]. Forested area in the Amazon Basin, where the largest rainforest on Earth is found, could be reduced in approximately 50% by 2050. [6–8].

While most deforestation occurs in the tropics, non-tropical forests are likely to suffer new deforestation pressures as the climate warms and areas which were previously too cold become suitable for agriculture [9, 10].

Assuming recent rates of human population growth are maintained until the end of the century, the Earth’s population will approach 10 billion around 2100. With current population to agriculture density of ∼ 147 people per km^2^, to meet the same quantity of food availability as present day, with no increases in productivity through technological advances, by 2100 agricultural areas would have to be increased by 43% [1].

Deforestation can impact climate on local and global scales by changes in the energy, mass and momentum fluxes between climate subsystems energy reservoirs. Deforestation is also associated with CO_2_ emissions, as crops and marginal lands that usually replace trees after land clearing tend to hold less carbon per unit area than forests [11, 12]. The radiative forcing associated with an increase in atmospheric CO_2_ is, from a climatic perspective, the most important biogeochemical impact of deforestation. Increases in CO_2_ also have the potential to affect climate by altering transpiration rates, due to CO_2_ increased water use efficiency reducing stomatal conductance and increasing plant growth [13–15].

The biogeophysical impacts of deforestation most pertinent to climate are changes to surface albedo, evapotranspiration (ET) and surface roughness length [16]. Croplands and pastures tend to have higher albedo than forests, which causes them to absorb a smaller fraction of the incoming solar radiation. Trees tend to have deeper rooting depth than crops and grasses such that tree removal implies a decreased ET and associated reduction in latent heat flux [12, 14, 17], ET can also be reduced through the reduction in canopy capture following deforestation, as well as from reduced turbulence associated with a lower aerodynamic roughness length and colder temperatures. For large-scale land cover change the alterations in ET could influence cloud formation potentially impacting atmospheric albedo and atmospheric longwave absorption [12].

In previous modelling efforts, the net temperature response to deforestation, to a large extent, is determined by the magnitudes of these opposing warming (higher atmospheric CO_2_ and lower latent heat flux) and cooling (increased albedo) effects (for some examples: [11, 12, 18–21]). The albedo-related cooling is particularly important at mid to high latitudes, where the presence of snow exacerbates the differences in reflectivity between forests and fields [11, 12], while the warming due to decreases in latent heat flux has a greater impact at low latitudes where the absolute changes in ET are larger [12, 22, 23].

Most modelling studies so far have analyzed the response to large-scale land cover change. In some, deforestation was global or performed over whole latitude bands [12, 18, 20, 24, 25] while others simulated global historical anthropogenic deforestation [21, 26, 27]. In general terms, these past simulations show that the temperature response of high latitude deforestation is still dominated by the albedo effect, resulting in a cooler climate. That is, while deforestation causes atmospheric CO_2_ concentrations to increase, the increment in albedo is enough to counteract greenhouse gas warming and yield a reduced surface air temperature (SAT). This cooling is global, and centered over the deforested areas [12, 22, 24]. Global temperature changes associated with mid latitude deforestation follow the same trend as for the high latitudes but with temperature changes of smaller magnitude [12]. Contrary to the cooling seen in the mid and high latitudes, simulated low latitude deforestation yields a warmer climate, with the increase in temperature attributed to the reduction in ET, and increased atmospheric CO_2_, which dominates the temperature signal [25, 28]. Some studies have noted that a reduction in cloud cover, and hence, reduced atmospheric albedo over deforested regions was an important contributor to the modelled warming [12]. There have been indications from satellite based observations [29] and modeling [23, 30] efforts that the temperature response is dependent on the scale and location of land cover change. According to these studies, in many high latitude and mid latitude areas deforestation would result not in cooling but in net warming or no significant change as the CO_2_ and ET induced energy gain overtakes the albedo induced losses.

Here we use a global climate model of intermediate complexity, with a coupled carbon cycle model, to determine to what degree the scale of deforestation may influence the climate system’s response to high, mid and low latitude deforestation. This is done by a series of experiments, where deforestation fractions range from 5%-100% of the tree covered area over these distinct latitude bands. The simulations are conducted from 2011 to 2100 with CO_2_ emissions based on the IPCC A2 scenario [31].

## 1 Model Description

The University of Victoria Earth System Climate Model (UVic ESCM) version 2.9 is an intermediate complexity climate model with horizontal resolution of 1.8° (meridional) *X* 3.6° (zonal). It is composed of a vertically integrated energy-moisture balance atmospheric model, a dynamic-thermodynamic sea-ice model, a continental ice dynamics model, and version 2.2 of the Geophysical Fluid Dynamics Laboratory (GFDL) Modular Ocean Model (MOM2). The MOM2 is a general circulation ocean model with 19 vertical layers. The terrestrial carbon model is a modified version of the MOSES2 land surface model and the TRIFFID dynamic vegetation model [32, 33]. Ocean inorganic carbon is based on the OCMIP abiotic protocol. Ocean biology is simulated by an ecosystem model of nitrogen cycling [34, 35]. Water, heat and carbon are conserved between model components with no flux adjustments. Cloud cover is set at a constant in the UVic ESCM. It has been shown that that large-scale deforestation may influence cloud cover and have an effect on the climate [12], however uncertainties exist in the change of cloud cover due to deforestation [29, 36, 37]. Precipitation is a function of relative humidity and not influenced by the fixed cloud cover. A full description of the atmospheric, oceanic, and sea ice models are in [38], while the land surface scheme and dynamic vegetation model are described in [32, 33].

### 1.1 Vegetation Model and Land Surface Scheme

TRIFFID defines the state of the terrestrial biosphere in terms of soil carbon, and the structure and coverage of five plant functional types (PFT), broadleaf trees, needleleaf trees, C_3_ grasses, C_4_ grasses and shrubs within each grid cell [32]. Using a carbon balance approach, TRIFFID determines the change in areal coverage, leaf area index and canopy height, as a result of net carbon fluxes calculated by the MOSES 2 land surface scheme. MOSES 2 recognizes the five PFTs used by TRIFFID, plus four non-vegetation types, bare soil, urban areas, land ice and inland water. Based on a photosynthesis-stomatal conductance model, plant respiration and photosynthesis are dependent upon climate and atmospheric CO_2_ [39]. Through this, the response of vegetation to climate occurs via climate-induced changes in the vegetation to atmospheric fluxes of carbon [32]. In each 1.8°*X* 3.6° grid cell, changes to land coverage through time is determined by a dynamic competition between the different PFTs. This is based on the Lotka-Volterra approach and a tree-shrub-grass dominance hierarchy. TRIFFID also allows agricultural areas to exist. These areas are defined as croplands and are treated as grass PFTs for determining their biogeochemical and biophysical behaviors.

Due to the non-linear character of the Lotka-Volterra equations used for the competition algorithm, there exists a possibility for rapid loss of vegetation species if the land-use disturbance is large enough to trigger the requisite scenario. This scenario can produce rapid increases or decreases in the abundance of a species [40].

Soil carbon pools are increased through litterfall, and reduced by heterotrophic respiration Litterfall is calculated as an area weighted sum from each PFT, and is dependent upon the degree of the land disturbance and competition between PFTs. Respiration is determined by the soil carbon content, a q_10_ soil temperature equation, and a piecewise linear soil moisture function [32]. In general terms this means that higher NPP results in an increase in the rate with which carbon is added to the soil with higher soil temperatures and moisture resulting in increases in respiration and hence in the rate of soil carbon decrease. Soil temperature, moisture and carbon content represent values from the model’s single one meter deep layer.

## 2 Experiments

For all experiments, the model is integrated from equilibrium at year 1800 to year 2000 forced by historical CO_2_ emissions from combustion of fossil fuels and land-use change [41, 42]. For the period between 2001 and 2100 simulations are forced by CO_2_ emissions from the IPCC A2 scenario [31].

**Fig 1:**
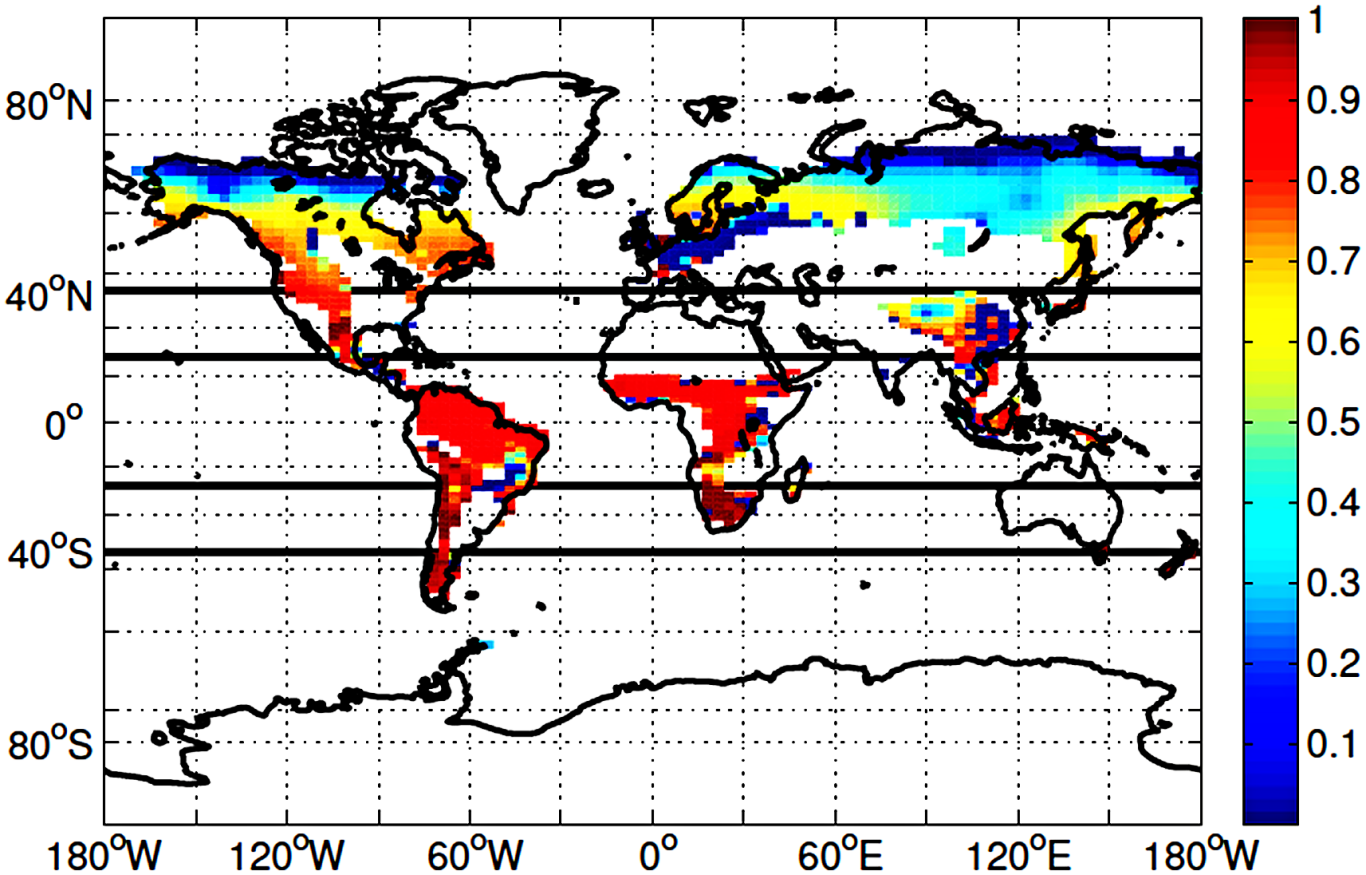
2010 Forest Coverage. Coverage is represented as fractional amount of broadleaf and needleleaf trees in each grid cell. Latitude bands designated by black lines. 40°N and above for the high latitudes, 20-40°N and 20-40°S for the mid latitudes and 20°S to 20°N for the low latitudes.

Deforestation experiments cover the period between 2010 and 2100. Deforestation is simulated separately in three bands: the area northward of 40°N (high latitudes), the areas between 20°N to 40°N, and 20°S to 40°S (mid latitudes) and the area between 20°S to 20°N (low latitudes) (Fig 1). While the adoption of latitudinal bands is our best attempt to represent impacts of deforestation on different environments, it must be noted that, as TRIFFID only contains five PFTS, the type of vegetation cover present in each latitude band is a very simple and incomplete representations of existing biomes, which are not only more complex but also distributed over the landscape space in complex patterns dictated by many other factors in addition to latitude.

At the start of 2010, all experiments have the same crop area distribution based on [2]. The vegetation is specified by a land cover data set [43]. All results are compared to a control run where the crop area fraction remained fixed at the 2010 distribution. In the deforestation experiments crop area fraction is increased by different amounts in order to generate arbitrary deforestation ranging from 5% to 100% of the total forested area of the three different latitudes at 2010. The land cover change is performed in a single step at the start of 2011 by substituting trees with crops.

**Fig 2:**
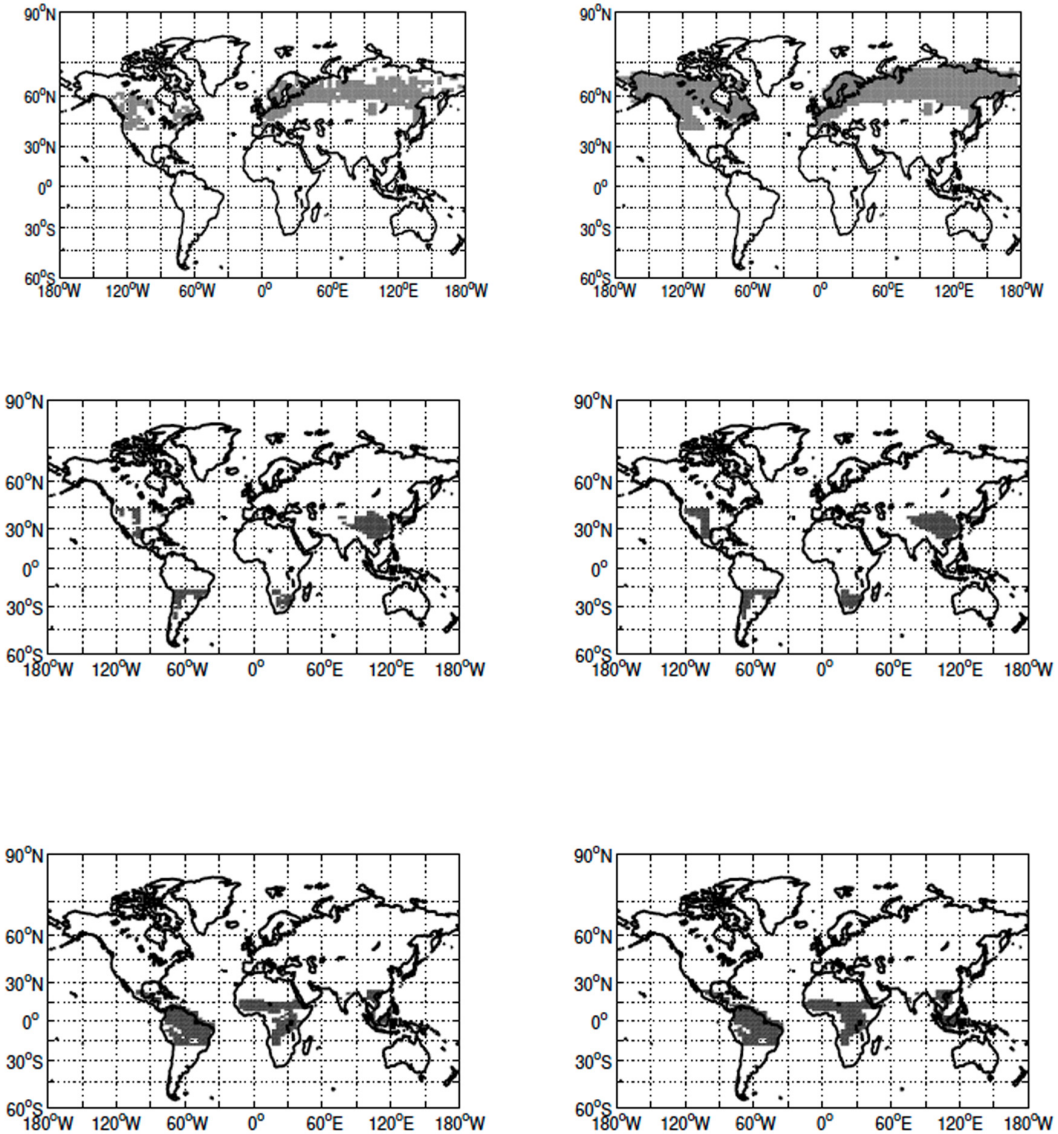
Grid cells eligible for deforestation in the high (top row), mid (middle row) and low latitudes (bottom row) in the non 100% simulations (left column) and 100% simulations (right column).

In all but the 100% deforestation scenario only grid cells that contain both crops and forests are defined as eligible for deforestation (Fig 2). In these simulations deforestation is performed by reducing the forest cover by a fixed amount in all eligible grid cells. The rationale is that experiments should simulate, as well as the coarse spatial scale of the model allows, land cover change resulting from an expansion in agricultural areas. In the 100% scenario, any grid cell with forests was deemed eligible for deforestation. There is no 75% deforestation simulation for the high latitudes, as the requirements for deforestation did not allow sufficient grid cells to be used to reach the required forest loss.

The expansion of croplands in the model follows a hierarchy where grasslands are converted to crops, before shrubs and trees. The result is that eligible grid cells that contain grasslands and shrubs prior to deforestation have these fractions converted to crops as well at the start of 2011. It should be noted that in TRIFFID crops and grasslands have identical biogeochemical and biogeophysical characteristics. The only difference is that grasslands can be outcompeted by other plant functional types while the crop distribution is prescribed. This means that all areas converted to crops in 2011 remain as such until the end of the experiments.

## 3 Results

After the initial disturbance the remaining forest cover is free to change according to the vegetation model’s response to climate. In presenting and discussing our results, experiments are classified according to their initial arbitrary deforestation fraction. For example, the 5% experiment refers to the simulation in which 5% of the forest cover was removed instantaneously at the start of 2011. Average SAT, soil temperature and P-E values for the 2090-2100 decade and their 95% confidence intervals are presented in Tables 1 and 2.

**Table 1:** Global means. Average (final) and 95% confidence margin of error (err) for last 10 years of the experiments from the high- (H), mid- (M) and low-latitude (L) deforestation scenarios. Lines labeled *5-100* refer to initial deforestation fraction. Errors are estimated based on a *t* confidence interval for the mean.

Global Surface Air Temperature						
	H err	H final	M err	M final	L err	L final
*5*	0.001	-0.044	0.003	-0.02	0.001	0.031
*15*	0.002	-0.047	0.004	0.015	0.002	0.162
*25*	0.002	-0.075	0.004	0.02	0.002	0.166
*45*	0.002	-0.016	0.004	-0.008	0.001	0.14
*75*	-	-	0.005	-0.058	0.001	0.06
*100*	0.005	-0.377	0.006	-0.077	0.001	0.044
Global Soil Temperature						
	H err	H final	M err	M final	L err	L final
*5*	0.001	-0.067	0.001	-0.02	0.002	0.074
*15*	0.002	-0.069	0.002	0.041	0.003	0.302
*25*	0.002	-0.104	0.002	0.056	0.003	0.305
*45*	0.002	-0.216	0.002	0.02	0.003	0.269
*75*	-	-	0.002	-0.04	0.003	0.166
*100*	0.005	-0.498	0.003	-0.056	0.003	0.162
Global Precipitation—Evaporation * *e*^−7^						
	H err	H final	M err	M final	L err	L final
*5*	0.04	-0.09	0.05	-0.33	0.06	-0.24
*15*	0.06	-0.05	0.06	-0.24	0.07	0.76
*25*	0.07	-0.2	0.07	-0.39	0.08	0.55
*45*	0.09	-0.9	0.09	-0.95	0.09	-0.06
*75*	-	-	0.1	-1.62	0.1	-1.24
*100*	0.1	-1.9	0.1	-2.1	0.1	-2

**Table 2:** Local Means—Same as Table 1 for values over deforested area.

Local Surface Air Temperature						
	H err	H final	M err	M final	L err	L final
*5*	0.01	-0.14	0.01	-0.063	0.01	-0.3
*15*	0.01	-0.22	0.004	-0.055	0.01	-0.4
*25*	0.01	-0.28	0.004	-0.064	0.01	-0.5
*45*	0.01	-0.42	0.004	-0.108	0.01	-0.8
*75*	-	-	0.01	-0.174	0.01	-0.16
*100*	0.01	-0.9	0.01	-0.222	0.01	-0.19
Local Soil Temperature						
	H err	H final	M err	M final	L err	L final
*5*	0.02	-0.13	0.05	0.08	0.03	0.21
*15*	0.03	-0.08	0.07	0.36	0.05	0.75
*25*	0.03	-0.13	0.07	0.47	0.05	0.76
*45*	0.04	-0.3	0.07	0.44	0.05	0.7
*75*	-	-	0.08	0.38	0.05	0.57
*100*	0.03	-0.79	0.08	0.37	0.05	0.56
	-	-				
Global Precipitation—Evaporation * *e*^−6^						
	H err	H final	M err	M final	L err	L final
*5*	0.07	-0.09	0.2	-0.7	0.2	-0.6
*15*	0.08	-0.4	0.2	-1.3	0.2	-0.7
*25*	0.08	-0.6	0.2	-1.8	0.2	-1
*45*	0.1	-1	0.3	-2.6	0.2	-1.5
*75*	-	-	0.4	-3.4	0.2	-2.3
*100*	0.1	-0.7	0.4	-3.2	0.2	-2.3

### 3.1 Further forest loss

**Fig 3:**
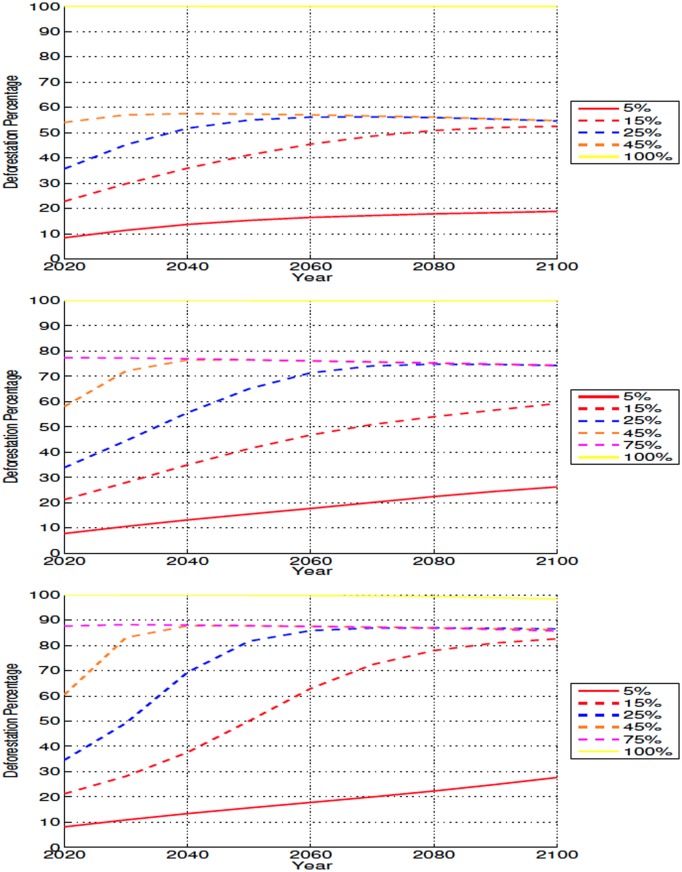
Annually averaged latitudinal forest coverage for high (top), mid (middle), and low latitude (bottom) deforestation simulations. Deforestation percentage is relative to the control run at the same time step.

With the exception of the 100% simulations, all deforestation scenarios, regardless of location, experience further loss of forests after the initial disturbance (Fig 3). In all experiments, the fraction of forest loss of the 25%-75% simulations tends to converge to around 50% in the high latitudes, 70% in the mid latitudes and 80% in the low latitudes. The 5% and 15% scenarios, continue to loose forests up to the end of the simulations. Pre-deforestation forest cover is identical for all experiments. The initial and continued forest loss percentages are based on this original, pre-deforestation value.

In all cases, the post-deforestation dieback is caused by further forest loss in the deforested bins, which in the more extensive deforestation scenarios tend to lose all of their trees. It is this loss of trees in the deforested bins which produces the converging trend observed in the 25%-75% simulations, as their forest coverage in the deforested bins is near identical. Some regrowth occurs in the non-deforested bins, where the forested fraction increases in relationship to the control for all simulations. This further loss of trees is not related to the dynamic vegetation model responding to climatic changes brought by deforestation but occurs because of the response of the competition algorithms adopted by TRIFFID to the large and rapid land cover change implemented at 2011. Following the large land-use change, and subsequent changes to climate, TRIFFID’s competition algorithm produced a continual loss of forests in the disturbed bins, with these forests being primarily replaced by shrubs. In that sense, the observed continuos loss of forest cover after deforestation are more akin to an external forcing to the simulations than to a response of the vegetation model to environmental change. Since our goal is to evaluate the climatic response to vegetation change and not vice-versa we feel that the continuous loss of forest resulting from this limitation of the competition algorithms do not invalidate our analysis and results.

### 3.2 Temperature and Moisture Response

The modeled temperature and carbon cycle responses to deforestation are intrinsically linked, complicating our choice of what to present and discuss first. Some readers might wish to come back to the temperature findings after reading the carbon cycle results (Section 3.3).

#### 3.2.1 High Latitudes

**Fig 4:**
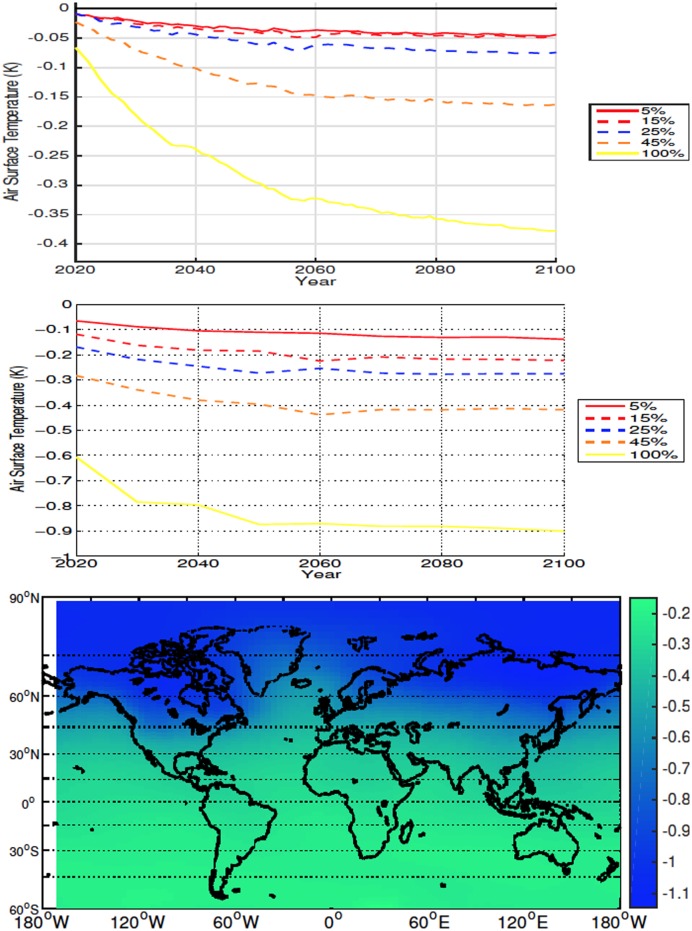
Annually averaged global air surface temperature anomalies for high latitude deforestation (Top). Annually averaged air surface temperature anomalies over deforested areas for high latitude deforestation (Middle). Air surface temperature anomalies at 2100 for the 100% high latitude deforestation simulation (Bottom). All anomalies are shown in K. Here, and in all other cases, anomalies are calculating by subtracting the annual value from the control simulation from the annual experiment value.

**Fig 5:**
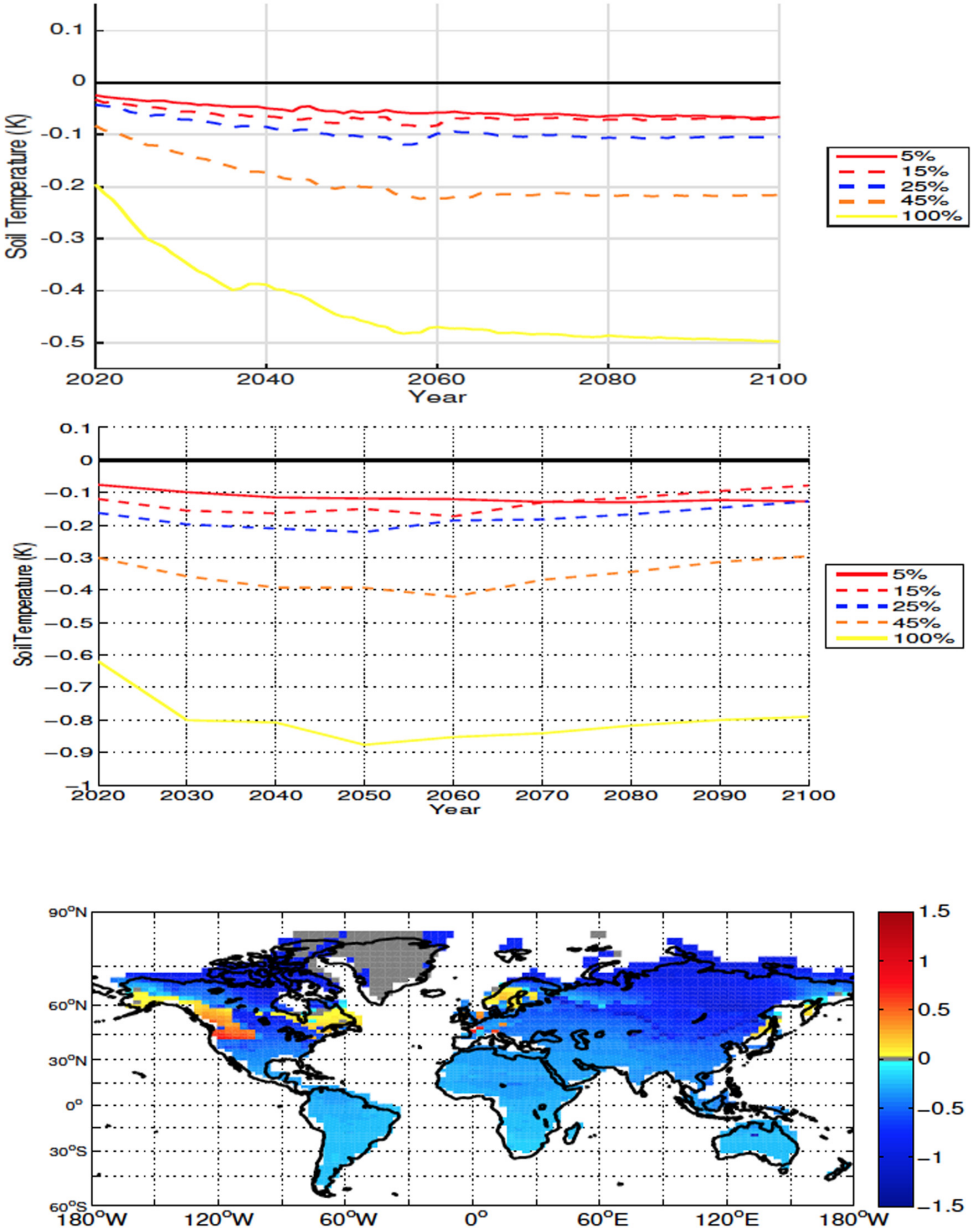
Annually averaged global soil temperature anomalies for high latitude deforestation (Top). Annually averaged soil temperature anomalies over deforested areas for high latitude deforestation (Middle). Soil temperature anomalies at 2100 for the 100% high latitude deforestation simulation (Bottom). All anomalies are shown in K.

For all simulations deforestation causes a reduction in global SAT, with the cooling being proportional to deforested percentage (Fig 4). The reduction in temperature is magnified at higher latitudes due to an increase in snow and ice cover and consequent increase in albedo. In the 100% scenario the average temperature change from 20°N to 40°N was -0.42K and the temperature change from 40°N and above was -0.78K. Lower atmospheric CO_2_ values are also responsible for some of the larger cooling seen in the 45% and 100% deforestation simulations (see Section 4). Deforestation also causes a decrease in global and local soil temperature (Fig 5—The term local refers to area weighted averages over the initially deforested grid cells), however soil temperatures become warmer in areas with large forest cover prior to deforestation (Fig 1). This warming is due to, similarly to what is observed more commonly in the mid- and low-latitude experiments, a decrease in sensible heat flux following deforestation. Soil temperatures show a similar trend to the SAT response

**Fig 6:**
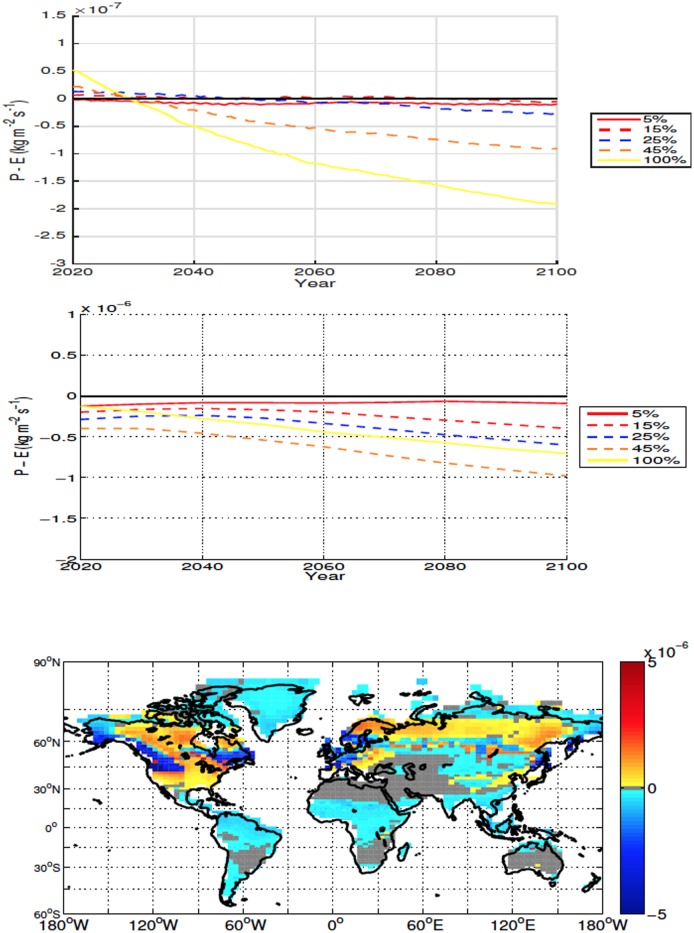
Annually averaged global precipitation minus evapotranspiration (ET) anomalies over land for high-latitude deforestation (Top). Annually averaged precipitation minus ET anomalies over deforested areas for high latitude deforestation (Middle). Precipitation minus ET anomalies at 2100 for the 100% high latitude deforestation simulation (Bottom). All anomalies are shown in kg m^−2^ s^−1^. Note that the global anomalies are adjusted by 10^−7^ while local ones are multiplied by 10^−6^.

Although the averaged global and local precipitation minus evaporation (P-E) point to drying, local drying is an order of magnitude larger than the global averages (Fig 6, top two panels). The areas with the largest drying occur in regions of increased soil temperatures. In these areas both ET and precipitation increase, however the increase in ET is larger than the increase in precipitation due to the enhanced soil temperatures. In the areas where conditions become wetter, there is also an increase of both precipitation and ET, however the increase in precipitation is larger. For all scenarios deforestation results in an overall drier climate over deforested areas (Fig 6, top two panels).

#### 3.2.2 Mid Latitudes

**Fig 7:**
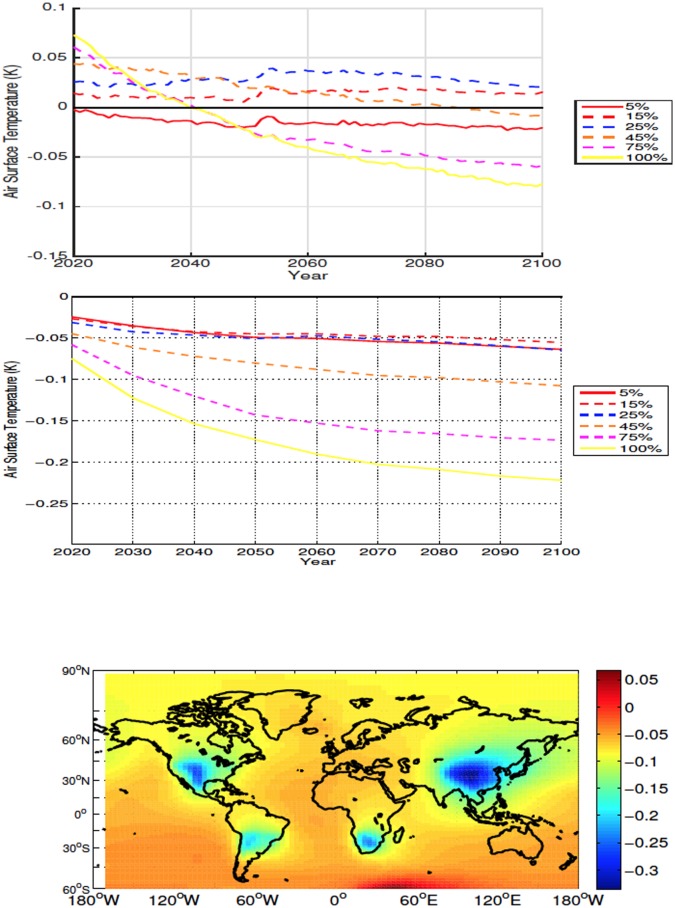
Annually averaged global air surface temperature anomalies for mid latitude deforestation (Top). Annually averaged air surface temperature anomalies over deforested areas for mid latitude deforestation (Middle). Air surface temperature anomalies at 2100 for the 100% mid latitude deforestation simulation (Bottom). All anomalies are shown in K.

Mean global SAT anomalies, while statistically significant by year 2100 (Table 1), are small and straddle zero K. At the end of the century the general tendency is cooling in the lower deforestation fraction with slight warming in the 15% and 25% scenarios. The larger input of CO_2_ into the atmosphere results in initial warming for deforestation of and above 45% (see Carbon Cycle section below). By mid-century albedo effects overcome the initial increase in greenhouse gas concentration and at the end of the simulation these scenarios exhibit very small SAT change or some cooling (Fig 7). The increase in local albedo dominates the temperature response over deforested areas which remain colder than the control even during periods where higher atmospheric CO_2_ cause mean global positive SAT anomalies (Fig 7).

**Fig 8:**
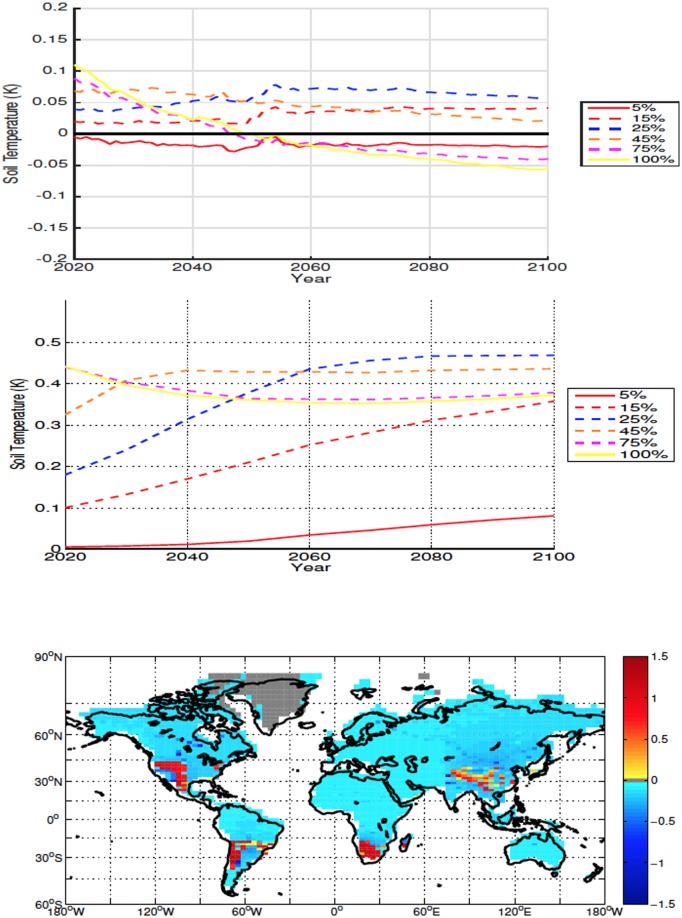
Annually averaged global soil temperature anomalies for mid latitude deforestation (Top). Annually averaged soil temperature anomalies over deforested areas for mid latitude deforestation (Middle). Soil temperature anomalies at 2100 for the 100% mid latitude deforestation simulation (Bottom). All anomalies are shown in K.

The globally averaged time series of soil temperature response shows a similar pattern to the SATs (Fig 8). In all simulations, average local soil temperatures over deforested bins are warmer than those of the control, with cooling occurring outside of the deforested areas (Fig 8). The initial local soil temperature response is proportional to the amount of deforestation. In most cases positive anomalies continue to increase, the exceptions being the 75% and 100% simulations which, due to reduced rates of energy absorption, resulting from increased albedo and outgoing latent heat flux, have a decreasing trend following deforestation.

**Fig 9:**
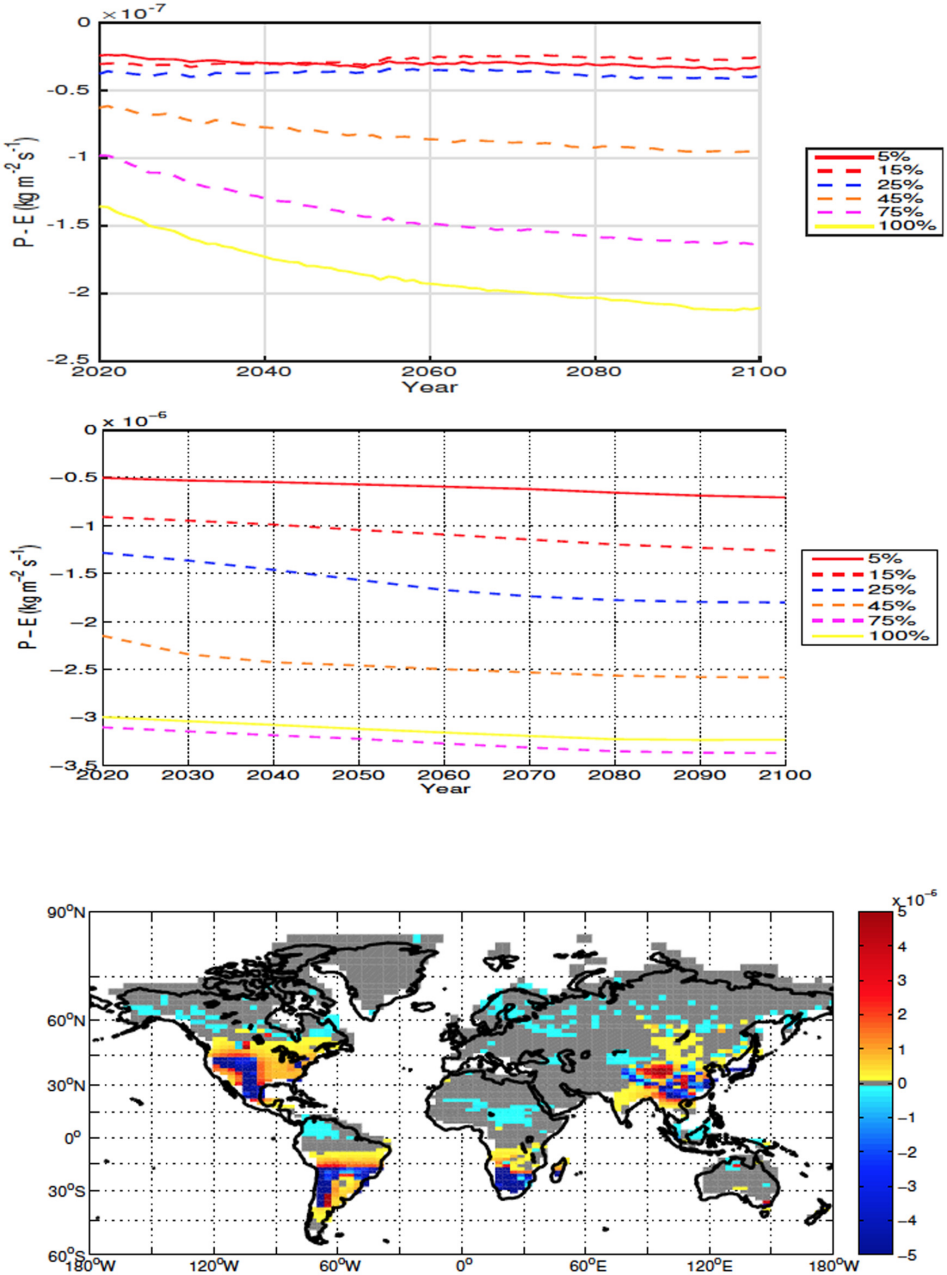
Annually averaged global precipitation minus evapotranspiration (ET) anomalies over land for mid latitude deforestation (Top). Annually averaged precipitation minus ET anomalies over deforested areas for mid latitude deforestation (Middle). Precipitation minus ET anomalies at 2100 for the 100% mid latitude deforestation simulation (Bottom). All anomalies are shown in kg m^−2^ s^−1^.

In all scenarios, deforestation leads to mean drying over land, with the decrease in moisture driven predominately by change over deforested bins (Fig 9). Drying over deforested areas tends to be proportional to the deforestation fraction, the exception being the 100% simulation which, although drier than the control, has slightly higher P-E values than the 75% scenario.

Mean ET and precipitation both increase over deforested areas, however in all simulations ET increases more than precipitation. Differing from the mean response, conditions are wetter in a significant number of deforested bins over Eastern Asia (Fig 9). While precipitation does increase, the positive P-E anomaly over these deforested bins is caused by a decrease in ET. The change in soil temperature over deforested areas in Eastern Asia also tends to be different from that of other areas. While the general response is warming, the temperature increase tends to be smaller and many bins in the area show cooler soil conditions after deforestation (Fig 8).

#### 3.2.3 Low Latitudes

**Fig 10:**
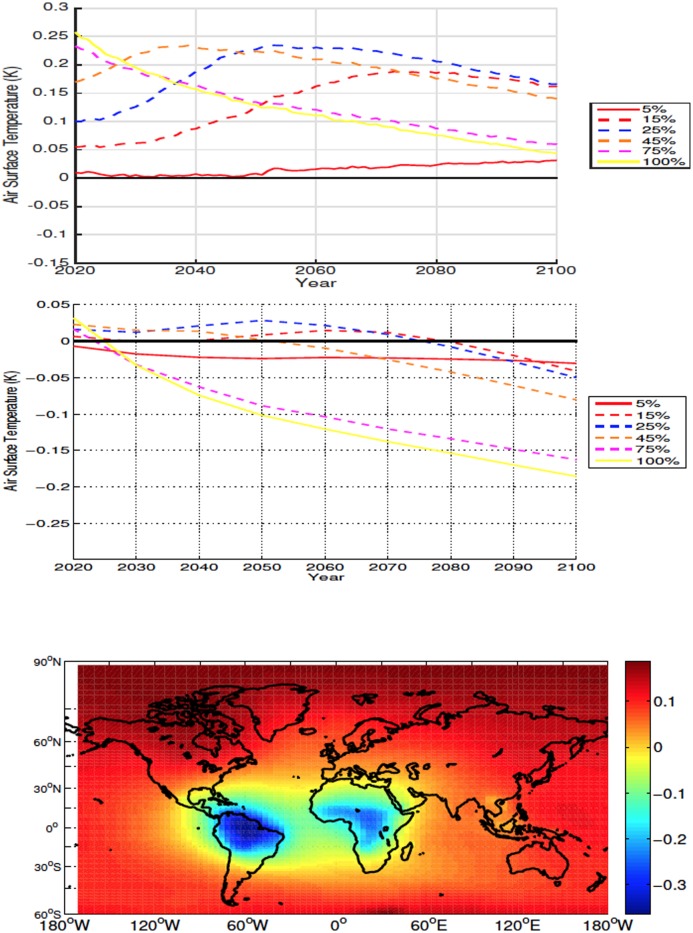
Annually averaged global air surface temperature anomalies for low latitude deforestation (Top). Annually averaged air surface temperature anomalies over deforested areas for low latitude deforestation (Middle). Air surface temperature anomalies at 2100 for the 100% low latitude deforestation simulation (Bottom). All anomalies are shown in K.

The global SAT response to deforestation of 15% and higher is a general increase followed by cooling (Fig 10). The magnitude and rate of initial warming tends to be proportional to the deforested area fraction and higher deforestation fractions tend to reach their peak positive anomaly sooner. Similar to the mid-latitude experiments, albedo effects start to overcome the initial CO_2_ driven warming but not to the point of generating negative anomalies (Fig 10). For the 5% scenario there is relatively constant warming associated with the progressive loss of forested area and addition of CO_2_ to the atmosphere seen in this experiment (see Carbon Cycle results below). SAT change over deforested bins is eventually dominated by the local increase in albedo and differs significantly from what is seen globally. At the end of the simulation all scenarions show negative local anomalies. As in the global case, the relative importance of albedo effects tend to be increase with deforested area (Fig 10).

**Fig 11:**
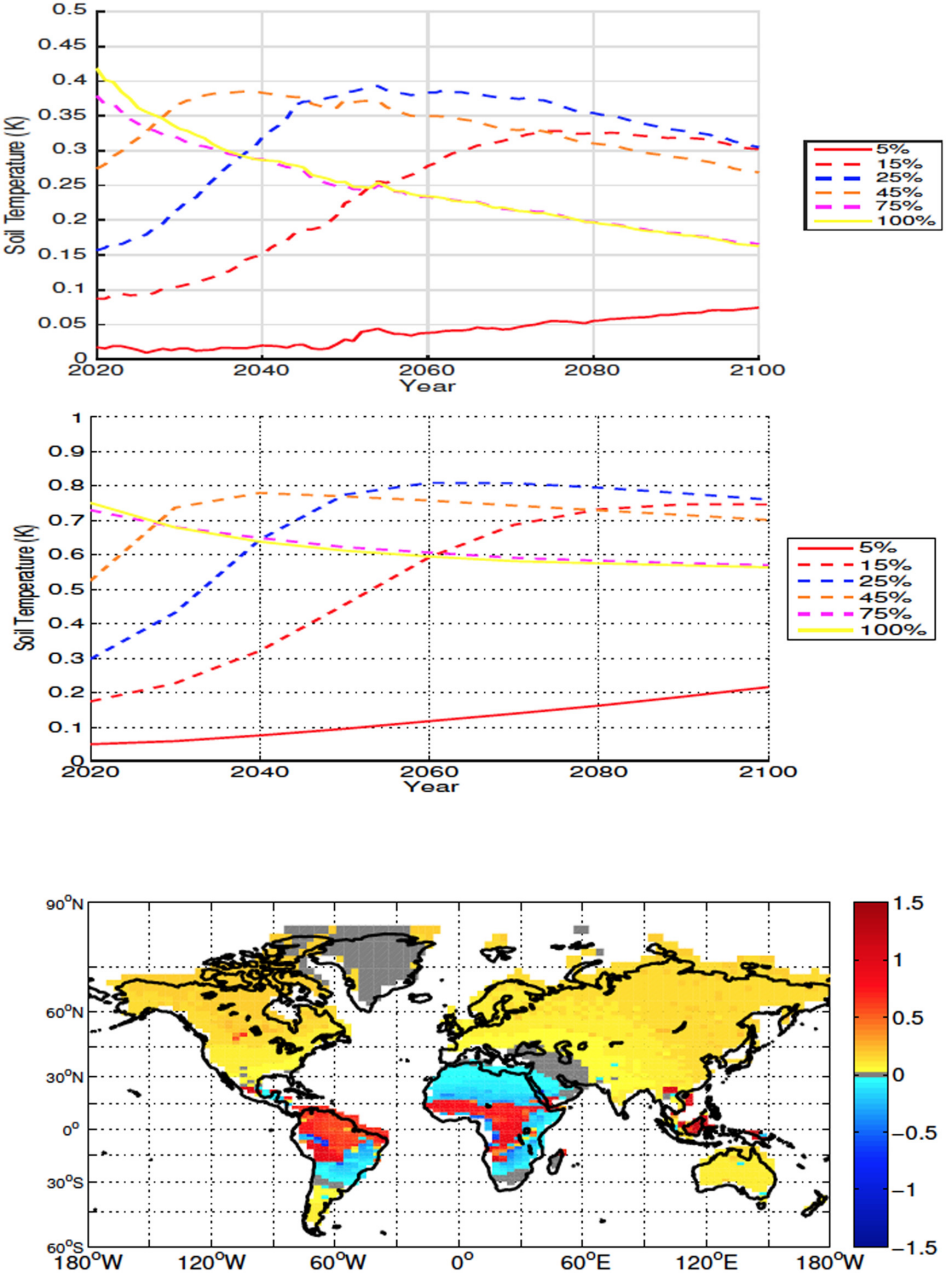
Annually averaged global soil temperature anomalies for low latitude deforestation (Top). Annually averaged soil temperature anomalies over deforested areas for low latitude deforestation (Middle). Soil temperature anomalies at 2100 for the 100% low latitude deforestation simulation (Bottom). All anomalies are shown in K.

The globally averaged time series of soil temperature response to deforestation exhibit a pattern similar to that of the global SAT but anomalies remain positive (Fig 11). The pattern of local soil temperature response over deforested bins is similar to the global response for the 5%-45% simulations. The 75% and 100% scenarios, similarly to what is observed at mid latitudes, show positive anomalies that decrease during to 21^*st*^ century resulting in soil temperatures that are still higher than the control but lower than those observed in the 15%-45% simulations.

**Fig 12:**
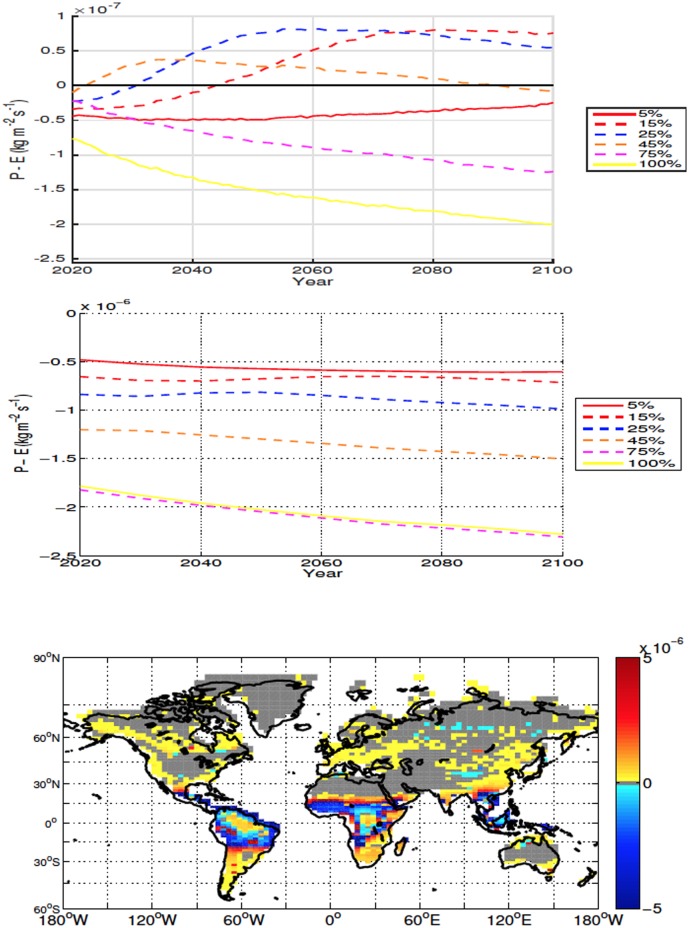
Annually averaged global precipitation minus evapotranspiration (ET) anomalies over land for low latitude deforestation (Top). Annually averaged precipitation minus ET anomalies over deforested areas for low latitude deforestation (Middle). Precipitation minus ET anomalies at 2100 for the 100% low latitude deforestation simulation (Bottom). All anomalies are shown in kg m^−2^ s^−1^.

The global moisture response over land over the low latitudes is less consistent between simulations than that of the high and mid latitude simulations, with most deforestation fractions changing between drier and wetter conditions during the experiment (Fig 12). In all experiments, conditions become drier over deforested bins and the P-E anomalies over these areas are an order of magnitude larger than those registered in the global response (Fig 12). Both ET and precipitation increase over deforested bins and drying occurs because the increase in ET overtakes the increase in precipitation. It is interesting to note that the local drying is usually more intense in the mid latitudes than in the low latitude simulations (Fig 12).

While the mean local response is drying, some areas in equatorial Africa and the Amazon become wetter after deforestation. Our simulations indicate that contrary to the areas that become wetter in the mid latitudes simulations, the positive P-E anomalies in the low latitude deforested bins are caused by an increase in precipitation and not decreased ET.

Compared to the mid latitudes, where the wetter conditions occur in areas of mixed warming and cooling soil temperatures, low latitude bins with positive P-E show no significant variation in soil temperature (Fig 11). From Fig 12 (bottom) it can be seen that there is an increase in P-E outside the deforested areas. This increase is not exclusive to the 100% deforestation shown, being seen in all scenarios. It is this increase in moisture in non-deforested areas, as well as the less pronounced decrease in moisture in the deforested areas for the 5%-45% simulations, that leads to an average global increase of moisture over land at various times in the simulations for the 15%-45% scenarios.

### 3.3 Carbon Cycle

#### 3.3.1 High Latitudes

**Fig 13:**
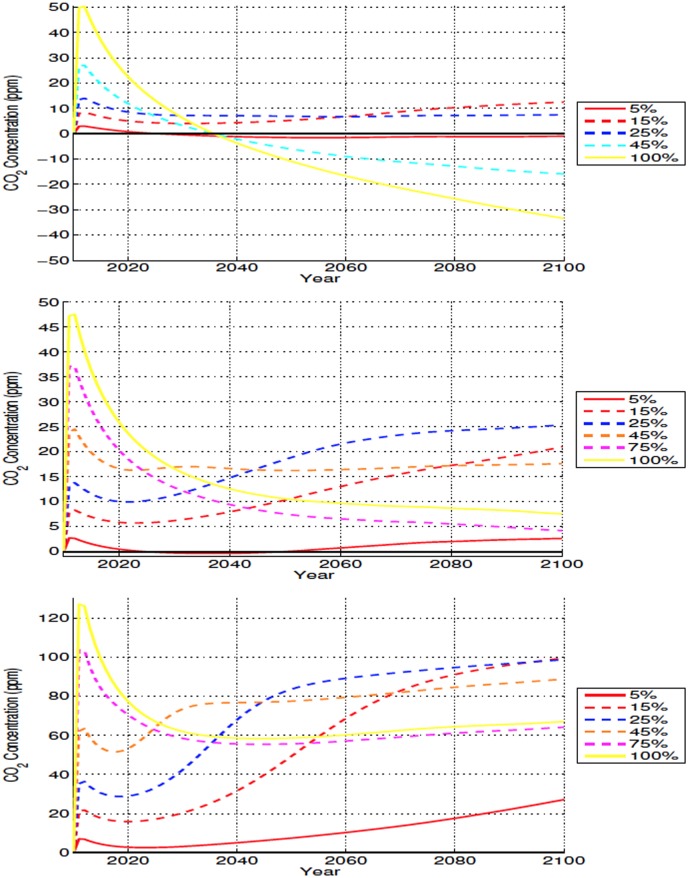
Annually averaged global CO_2_ concentration anomalies for high (top), mid (middle), and low latitude (bottom) deforestation. All anomalies shown in ppm.

All deforestation simulations show an initial increase in atmospheric CO_2_ relative to the control. The increase is proportional to deforested area and ranges from 3 to 50 ppmv (Fig 13, top). This expected increase in CO_2_ concentration is due to the release of carbon stored in the forests [44, 45]. Although the relative difference between the simulations and the control decreases in the first 10 years after deforestation, atmospheric CO_2_ values for the 15% and 25% deforestation experiments remain above those of the control during the whole simulation due to continued, competition algorithm induced, loss of forests leading to increased CO_2_ emissions. For the 45%-100% deforestation experiments however, the post-deforestation pulse reduction in CO_2_ is such that from about year 2040 onward these scenarios’ atmospheric CO_2_ concentrations are lower than those of the control (Fig 13, top).

**Fig 14:**
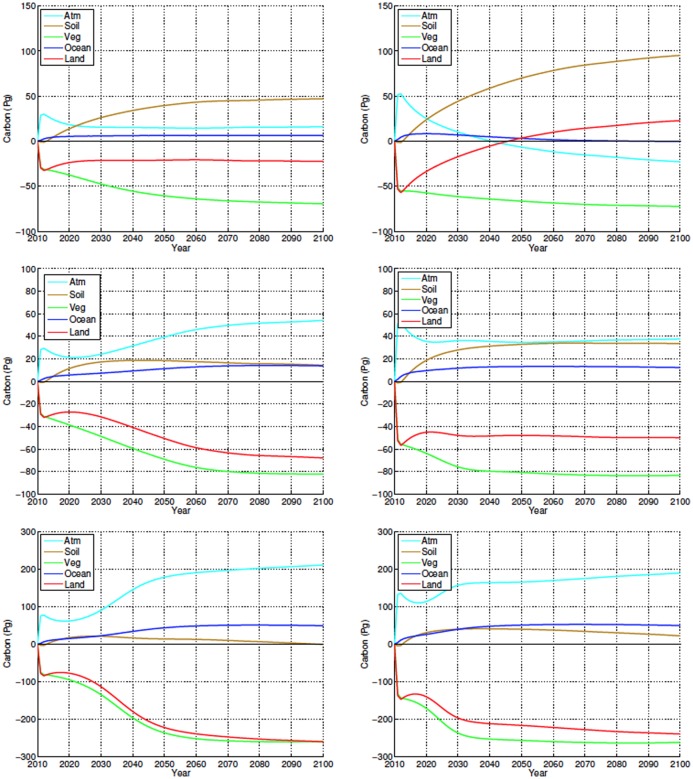
Temporal evolution of annually averaged carbon stock anomalies from different model components for high (top row), mid (middle row), and low latitude (bottom row) simulations for the 25% (left column) and 45% deforestation (right column) scenarios. All anomalies shown in Pg C.

In all simulations the behaviour of ocean carbon is similar to atmospheric CO_2_ and all simulations with a relative loss of atmospheric CO_2_ also show a relative reduction in ocean carbon (Fig 14, top row).

Atmospheric carbon closely mirrors the changes to land carbon—the sum of soil and vegetation carbon. CO_2_ levels are higher in experiments where deforestation leads to net loss of carbon by the land reservoir and lower in simulations where the removal of trees cause an increase in land carbon stocks (Fig 14). Deforestation always results in an decrease in vegetation carbon and an increase in the soil carbon pool. Some of this increase takes place over non-deforested areas where, for high- mid- and low-latitude deforestation, NPP increases due to changes in climate and the CO_2_ fertilization effect. The net change in the land carbon pool, and consequently the atmospheric CO_2_ response, is determined by the relative magnitude of these soil and vegetation carbon changes.

Ignoring the small drawdowns of the 5% experiment, deforestation of up to 25% results in losses in the land carbon pool and increase in atmospheric carbon (Fig 14). For the 45%-100% deforestation scenarios the increase in soil carbon overcomes the losses from vegetation carbon. In these simulations the land gains carbon at the expense of the atmosphere, where CO_2_ concentrations decrease (Fig 14).

The increase in soil carbon is related to a reduction in soil heterotrophic respiration due to colder and drier conditions and also to an increase in modelled net primary productivity (NPP) over deforested areas (not shown and caused by the higher modeled NPP of grasslands compared to forests in these latitudes). In the 5%-25% simulations the increase in soil carbon was larger in the non-deforested areas than in the deforested areas. We interpret this as an indication that climate played a larger role than alterations in NPP due to land cover change in the increase of soil carbon in these experiments. Deforested areas contribute to approximately 58%-70% of the increase in soil carbon in the 45%-100% deforestation scenarios, showing that in these experiments the higher NPP of croplands also played a role in accumulation of land carbon. Although croplands are usually associated with reductions in NPP, observations have found an increase in NPP after forest loss in high latitudes [46], and modeling efforts have found an increase due to higher grassland productivity and CO_2_-fertilization despite colder temperatures [24].

#### 3.3.2 Mid Latitudes

In all scenarios, deforestation produces a rapid increase in atmospheric CO_2_ (2 to 47 ppmv) as the carbon lost by vegetation due to deforestation makes its way into the atmosphere (Figs 13 and 14). This is followed again, in all scenarios, by a decrease in CO_2_ values. After this initial pulse, the behaviour of the simulations differs. The 5%-25% scenarios show increases relative to the initial post pulse value up to year 2100. CO_2_ concentrations remain near the post-pulse value for 45% experiment while the 75% and 100% scenarios, in similar fashion to the high latitude case, exhibit continuos reductions in atmospheric CO_2_ after the initial peak. Contrary to the high latitudes simulations these reductions in atmospheric CO_2_ are not large enough to generate negative anomalies by the end of the century.

All simulations experience a global increase in soil carbon that offsets a portion of the losses in land carbon caused by deforestation and dieback. Following a brief (about four years) initial decrease in soil carbon, all experiments show a relatively rapid increase of carbon in this pool up to about 2040 to 2060. The magnitude of the increase is dependent on the deforestation fraction and in all experiments this is accompanied by a reduction in atmospheric carbon (Fig 14).

For all experiments, both forested and non-forested bins contribute to the initial increase in soil carbon. Due to changes in climate and enhanced NPP caused by increased atmospheric CO_2_ [47], non-deforested bins maintain positive soil carbon anomalies over the duration of all experiments. As simulations progress, some scenarios experience eventual reductions in soil carbon, associated with loss of carbon in deforested bins. These losses are larger for the 15%-25% scenarios, (Fig 14) and are associated with increased respiration, due to warmer soil temperatures (Fig 8) and wetter conditions over their deforested bins when compared to those of the other scenarios (Figs 6, 9 and 12). The increase in NPP, as well as the enhanced drying and cooler temperatures over the deforested bins of the 75% and 100% experiments causes these areas to gain soil carbon continuously during the experiments. The large increase of soil carbon in the higher deforestation fraction experiments leads to the larger atmospheric CO_2_ drawdown seen in these scenarios.

Ocean carbon stocks are responding to atmospheric CO_2_ (Fig 14) as ocean carbon increases and decreases with the fluctuating CO_2_ values.

#### 3.3.3 Low Latitudes

As in the other latitudinal bands, the carbon lost by vegetation due to tree removal over the low latitudes enters the atmosphere, generating a rapid increase in CO_2_ concentrations proportional to the extent of deforestation (7 to 127 ppmv). Due to the larger quantity of trees removed in the low latitudes, this initial CO_2_ increase is higher than what is seen in the high and mid latitudes combined (Fig 13).

This increase is followed by a period of diminishing CO_2_, with the rate of decrease proportional to the deforestation fraction caused mostly by oceanic absorption of CO_2_. Atmospheric CO_2_ values in the 75% and 100% scenarios fall relatively quickly, and for the duration of the experiments, remain below those of the initial peak. This is not the case for the other scenarios, where by no later than the mid 21^*st*^ century, the atmosphere holds more carbon than it had at the peak of the post deforestation pulse (Fig 13).

The low latitudes had the same initial soil carbon response to deforestation as the high and mid latitudes, with a small initial decrease over the first few years followed by a rapid increase (Fig 14). As with the mid latitudes, the changes in soil carbon are explained by continuous increase in carbon density over non-deforested areas and a mixed response in the deforested bins. The largest soil carbon losses occur in the intermediary deforestation fractions, where soils are warmer and not as dry. Different from the high and mid latitudes, these losses resulted in periods where some intermediary fractions, like the 25% scenario, exhibited global changes soil carbon near zero. Again as seen in the high and mid latitudes, the experiments with the smaller soil carbon anomalies are the ones which show the largest concentrations of atmospheric CO_2_. Increases in NPP, relatively colder temperatures and drier conditions lead to large soil carbon gains by deforested bins in the higher fraction deforestation scenarios. Similarly to the mid latitudes, the deforested bins of the 75% and 100% simulations hold the majority of the global soil carbon by the end of the experiments.

The ocean carbon response to low latitude deforestation is very similar to the high and mid latitudes, where ocean carbon to a certain degree mirrors atmospheric carbon. In the low latitudes we do not see the same level of post initial pulse reduction in atmospheric carbon as was observed in the high and mid latitudes, and the ocean carbon also shows this, as there is less variation in total ocean carbon between the simulations, due to the oceans slower response to atmospheric carbon change (Fig 14).

## 4 Discussion

The use of a model of intermediate complexity in the study of deforestation has some drawbacks, among the more evident, the lack of a cloud response, which is expected to play a larger role in low-latitude deforestation. Still due to computational and time constraints, the large number of experiments required by the project could only be conducted with this kind of model.

### 4.1 High Latitudes

The lower temperatures observed in our 5%-25% deforestation experiments are in agreement with previous modelling studies where the albedo effect outweighs the increase in CO_2_ for high latitude deforestation [12, 22, 24]. Differently from these earlier results, our cooling is magnified in the 45%-100% scenarios by a decrease in atmospheric CO_2_ caused largely by an accumulation of soil carbon over deforested areas. By 2100 our 100% simulation produced cooling of approximately 0.4 K, with an average cooling over the duration of the simulation of 0.33 K. The average cooling is similar to what was found by previous experiments where a cooling of 0.25 K is observed [24], but at 2100 we obtained cooling that was only about half of what is modeled by other simulations [12]. In terms of albedo, we detected high-latitude increases ranging from 2.02% to 12.74% by 2100, with larger deforestation events producing higher increases in albedo. This is higher than the 10.7% increase of high latitude albedo at 2100 obtained by earlier efforts [12]. When global albedo is considered, our average increases of 0.01 to 0.04 are smaller than what was observed by other experiments that reported a global average increase of 0.07 [24]. It is likely that the discrepancies between our results and these past efforts are at least partly due to differences in the selection of the latitudinal ranges used by the different experiments [12, 24].

Earlier simulations of complete deforestation in areas above 45°N caused an increase in soil carbon over deforested areas [24], but in these experiments the extra soil carbon was not enough to compensate for the loss of biomass and litter carbon, leading to an overall loss of land carbon and a ≤ 5 ppm increase in atmospheric CO_2_ [24]. Another set of previous experiments, while not reporting changes in soil carbon, find a similar (5 ppm at 2100) increase in atmospheric CO_2_ in their 100% high latitude deforestation simulations [12]. It must be noted that one of these experiments did not account for anthropogenic emissions [24], which are considered by the other [12].

In a managed forest, clear cutting has been shown to reduce soil carbon [48]. At particular temperate zone sites conversion of forests to cropland resulted in about a 32% ±20% decrease in soil carbon and the conversion of grassland to forest caused soil carbon *reduction* of about 7%± 23% [49]. Even our low-end deforestation scenarios result in land cover change at much larger spatial scale than those analyzed by these observational studies [48, 49] and comparison with our results should be made with caution.

The initial surface air and soil cooling of the 45%-100% experiments are markedly larger (Figs 4 and 5) than those of the other simulations. It is this rapid albedo-related cooling, and the associated slow down of soil respiration, which lead to the larger retention of carbon over land in these scenarios. As the surface albedo and atmospheric CO_2_ begin to change, they influence the surface energy balance, which in turn effects the soil temperatures. In the case of the high latitudes, the resultant magnitude of the changes to the energy balance that lead to the decrease in soil temperatures.

**Fig 15:**
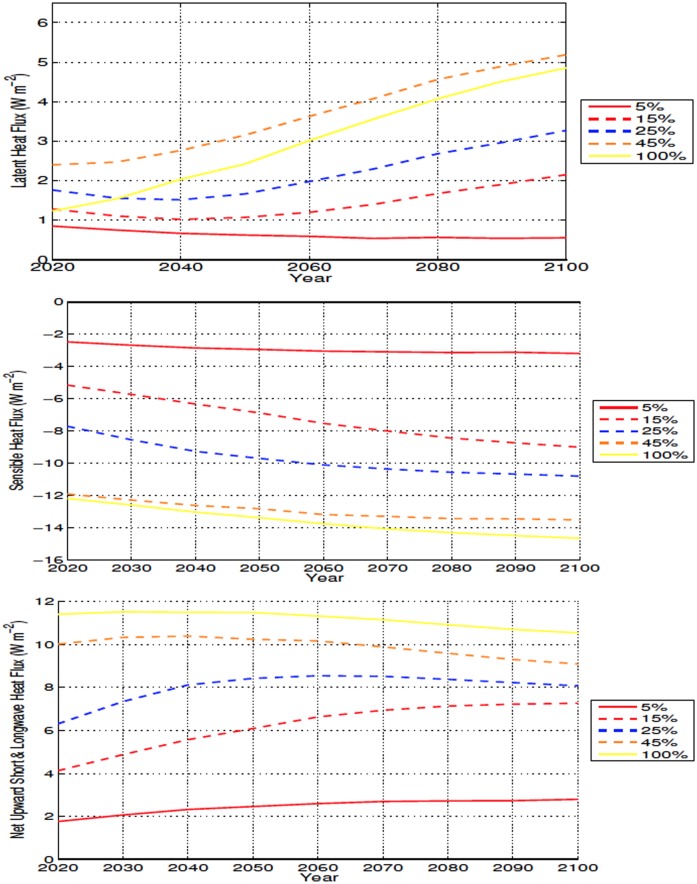
Annually averaged outgoing surface energy flux anomalies over deforested areas for high latitude deforestation. Energy fluxes shown as latent heat (Top), sensible heat (Middle), and net surface radiation (Bottom) All anomalies shown in W m^−2^.

Removal of trees shortens the roughness length and causes a reduction in outgoing sensible heat flux, a warming effect, over deforested bins. At the same time, deforested areas—due to an increase in modeled evaporation—experience increases in the outgoing latent heat flux and a decrease in shortwave absorption. The final result is a reduction in net incoming energy and soil cooling (Fig 15). In a similar way, high-latitude deforestation has been observed to cause local cooling despite a decreased outgoing sensible heat flux [50]

The energy budget is balanced by the equation:

$$\begin{array}{c} R_{n}=LE+H+G \end{array}$$ (1)

Where *R*_*n*_ is the net surface radiation, *LE* is latent heat, *H* is sensible heat, and *G* is the ground heat flux. By increasing the surface albedo, and latent heat, the decreased surface skin temperature contributes to the reduced soil temperatures.

The reduction in soil moisture, while less important in explaining the initial accumulation of soil carbon, is likely exerting a larger influence after about 2060, when soil carbon continues to increase (albeit at slower rates) in spite of increasing soil temperature over deforested areas (Figs 5 and 14). In Fig 6, an interesting result is shown where the drying in the 100% simulation isn’t the largest, compared to the temperature and carbon response, where the 100% scenario produces the most extreme changes. The increased moisture is likely due to the larger area used for 100% deforestation, with more snow covered areas and more cooling resulting in less ET. This result can also be observed in (Fig 15) where the latent heat flux of the 100% simulation is less than the 45% and 50% scenarios for the majority of the simulation.

Evidently, we don’t make the claim that our findings justify large scale high latitude deforestation as a means of carbon sequestration. Nevertheless, our results point to the complex interaction between soil carbon dynamics and climate and the significant role this interaction plays on the modelled climatic response to land cover change.

### 4.2 Mid Latitudes

The SAT cooling seen in the 100% mid latitude deforestation scenario is in general agreement with previous studies [12, 25], albeit the 0.077 K reduction in temperature at 2100 recorded by our experiments is larger than the 0.04 K cooling produced by comparable simulations [12]. Our experiments generated albedo increases of 1% to 5%, within the range range of earlier efforts that found increases of 4.7% to 5% [12].

By 2100 the 75% scenario has near identical forest loss as the 25% and 45%. However, the 75% and 100% experiments show higher initial albedo increase, as well as reduced atmospheric CO_2_ through increased soil carbon, driving further global cooling. The rapid increase in albedo in the 75% and 100% scenarios effect the soil temperatures as well, which decrease over the first 60 years, before the temperature anomalies begin to trend upwards again. This points to the importance of the magnitude of the initial disturbance on modelled climates. Although the 25%-75% scenarios all reach similar forest loss by 2100, and display converging albedo values, the larger initial land use change results in higher initial albedo values which allow the 45%-75% simulations to reach lower temperatures despite having final land use change fractions similar to the 25% simulations.

**Fig 16:**
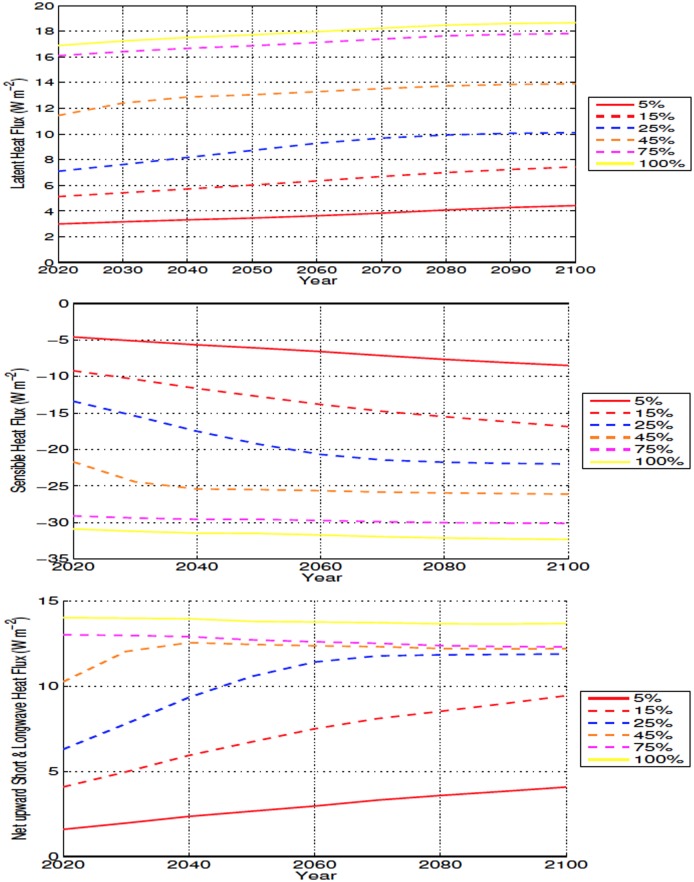
Annually averaged outgoing surface energy flux anomalies over deforested areas for mid latitude deforestation. Energy fluxes shown as latent heat (Top), sensible heat (Middle), and net surface radiation (Bottom) All anomalies shown in W m^−2^.

In the mid latitudes, the local response to deforestation differs compared to the high latitudes. The increase in ET leads to more drying than what is observed in the high latitudes (Figs 6, 9, 15 and 16). This is also accompanied by increased soil temperatures in the mid latitude deforested bins, opposed to the local response to high latitude deforestation (Figs 5 and 8). Soil temperatures increase, despite the added cooling from increased modelled outgoing latent heat and albedo, primarily due to the decreased roughness length following deforestation. Decreased roughness lengths lead to less outgoing sensible heat, which becomes the dominant driver in local soil temperatures for these experiments. Following deforestation the outgoing sensible heat flux (Fig 16) decreases more than at the high latitudes, resulting in a higher retention of heat in the soils, and thus higher soil temperatures.

Sensible heat flux in the UVic ESCM is represented by:

$$\begin{array}{c} SH=\rho C_{D}U\left(T_{s}-T_{a}\right) \end{array}$$ (2)

Where *SH* is sensible heat, *ρ* is the density of air, *U* is the wind speed, *T*_*s*_ is the soil temperature, *T*_*a*_ is the SAT and *C*_*D*_ is the Dalton number given by:

$$\begin{array}{c} C_{D}=k^{2}{\left(ln\frac{z}{z_{0}}\right)}^{-1}{\left(ln\frac{z}{e^{-2}z_{0}}\right)}^{-1} \end{array}$$ (3)

Where *k* is the Von Karman constant, *z* is a reference height and *z*_0_ is the aerodynamic roughness length [21, 51].

In the simulations, deforestation reduces *C*_*D*_ because the grassland *z*_0_ is smaller than the forest *z*_0_. This change in *C*_*D*_ is what inevitably reduces *SH*, as the other terms in the equation act towards increasing *SH*. Decreasing roughness length over the mid latitude deforested bins reduces the surface’s ability to lose sensible heat, resulting in a net soil temperature increase over these areas.

The accumulation of global soil carbon is driven by the increase in NPP and a decrease in soil respiration. The change in soil carbon accumulation is intrinsically tied to the change in soil temperature and moisture. Globally, all deforestation fractions led to drier conditions over land, and global soil temperatures tend to decrease, the exception being the 15% and 25% scenarios (Figs 9 and 8).

The opposing effects of warming and drying trends determines the exchange of carbon between soil and atmosphere. The 15% and 25% scenarios experience the largest local warming, however do not experience the same level of drying as is seen in the 45%-100% scenarios. The 45%-100% scenarios also experience less warming then the 15% and 25% scenarios, leading to reduced respiration in the 45%-100% cases and larger drawdown of atmospheric CO_2_. This results in larger soil carbon quantities and less atmospheric CO_2_ (Figs 13 and 14). The increase in CO_2_ seen in the 5%-25% simulations is linked to the less rapid loss of forests than was seen in the 45%-100% scenarios, resulting in less albedo cooling, and a slower release of CO_2_ over time. The warmer global soil temperatures of the 15% and 25% scenarios are due to the higher surface air temperatures, where increased CO_2_ concentrations, as well as lower surface albedos than the 45%-100% scenarios, highlighting the complex interactions which take place in determining local and global temperatures.

Similar to the high latitudes, some mid latitude deforestation simulations differed from observational studies where deforestation led to a decrease in soil carbon due to increased respiration [48, 52]. In our experiments the 45%-100% simulations resulted in local soil carbon increase, which is in agreement with observations that indicate that the conversion of grassland to forest causes a soil carbon reduction [49].

The response to deforestation in the mid latitudes shows the transition between high and low latitude deforestation where albedo change becomes a less dominant driver of temperature and changes to CO_2_ concentrations and the sensible heat flux play a larger role in local and global temperature response. The high latitudes experience cooling in every simulation almost instantaneously (Fig 4), whereas the presence of higher CO_2_, and decreased outgoing sensible heat flux overcome the albedo change and increased latent heat, resulting in longer lasting warmer conditions in the mid latitudes (Fig 7).

### 4.3 Low Latitudes

The initial air surface warming which occurs due to low latitude deforestation is in agreement with earlier studies [12, 24, 53]. The magnitude of the temperature change is not consistent with previous studies due to differences in the location and magnitude of deforested areas and CO_2_ concentrations. Our 100% simulation resulted in a 0.04 K global warming by 2100 while previous studies show values ranging from 0.4 K [24] to 0.7 K [12].

Our 3% to 10.8% albedo increase was larger than the 4.1% recorded for some experiments [12] while our average albedo increase of 0.01 to 0.02 was smaller than the increase of 0.04 seen in other simulations [24]. The local cooling observed in our study is seen, although not to the same extent, in some [12, 22] but not all [24] comparable experiments.

The difference between these studies is likely due to the different carbon and albedo responses, as well as some of the inherent differences in the models used. By 2100 our 100% scenario had an increased CO_2_ concentration of ∼ 66.5 ppm, while comparable experiments resulted in an increase of 199 ppm [12]. Another difference is that previous studies [12, 24] produced a reduction in ET, as well as a decreased atmospheric albedo from reduced cloud cover, both of which contribute to increased temperatures.

Satellite based studies show that that depending upon the scale of deforestation, cloud cover may not change and may even increase over disturbed areas [29, 36, 37]. This contradicts the above mentioned results [12, 24] and may lead to added cooling not accounted for in these modelling studies. One of these previous efforts [12] found that the net albedo change over the deforested regions was negligible as the increase in surface albedo was compensated by the decrease in atmospheric albedo, and suggests that cloud cover may play a major role in tropical climates.

Clouds cover is prescribed in the UVic ESCM and in our experiments the post-deforestation surface albedo increase is not compensated by a decrease in atmospheric albedo due to a reduction in cloud cover over deforested areas. This helps explains the local cooling registered by our simulations and lends support to the argument that a reduction in cloud cover is an important component of the modelled temperature response to deforestation in the tropics see in previous experiments.

Low latitude deforestation has been associated with a decrease in modelled total land carbon [24], and the same occurs in all of our simulations (Fig 14). The same authors [24] also see a reduction in soil carbon in the deforested areas, and increases in the non-deforested areas, which is observed in our 5%-45% scenarios, but is in disagreement with our 50%-100% experiments where we record an increase in soil carbon in all locations, regardless of land cover change. Soil temperatures were larger in the low latitudes, and conditions were wetter than the mid latitudes. It is likely this difference in climate that causes the larger reduction in local soil carbon, in the 5%-45% scenarios than the mid latitude cases. During the simulations the 5%-25% scenarios become wetter, relative to their initial drying following deforestation (Fig 12). This change in behaviour, as well as warmer soil temperatures (Fig 11), can account for the increased respiration relative to carbon drawdown, and hence the decreased local soil carbon in these experiments.

**Fig 17:**
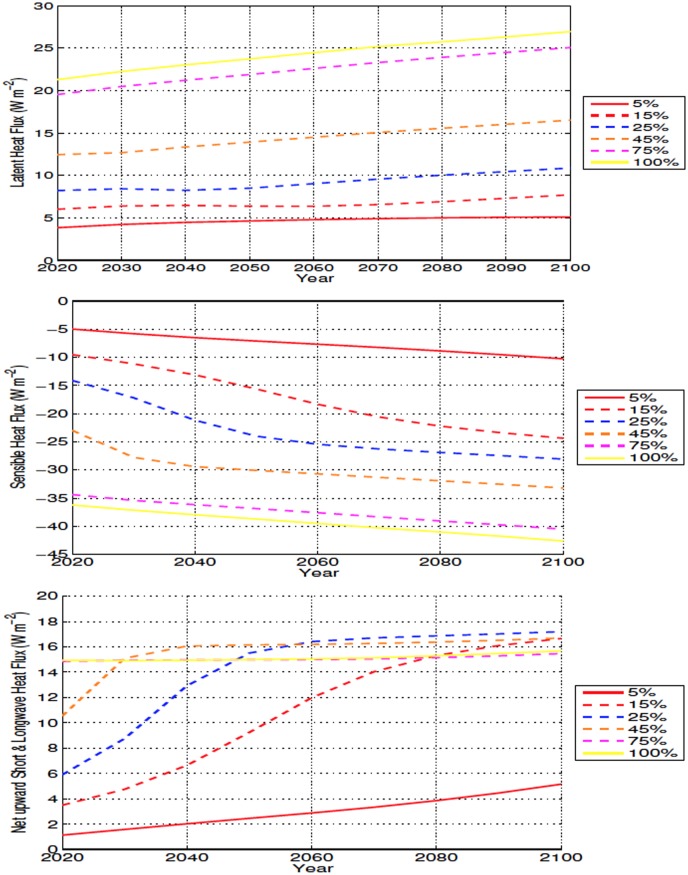
Annually averaged outgoing surface energy flux anomalies over deforested areas for low latitude deforestation. Energy fluxes shown as latent heat (Top), sensible heat (Middle), and net surface radiation (Bottom) All anomalies shown in W m^−2^.

Local soil warming occurs due to the same energy balance modifications seen in the mid latitudes, with higher temperatures related to a decrease in outgoing sensible heat flux that overcomes the increase in outgoing net radiative and latent heat fluxes (Fig 17). The higher local soil temperature anomalies present in the low latitudes, occur due to the larger reductions in outgoing sensible heat, as well as increased rates of change in the energy budget of the soils, than what is seen in the mid latitudes (Figs 16 and 17). The increase in outgoing latent heat flux and reduction in sensible heat flux, also explains the lower SATs over deforested areas in the low latitudes (Fig 10).

The direction of the soil carbon response of our 5- 45% low latitude simulations are in agreement with some observations [48, 52] but differ from what has been described by other [49]. Although NPP increases due to the forest-to-cropland conversion, this is not enough to overcome the changes in respiration, where consistently higher soil temperatures, as well as less drying than is observed in the mid latitudes (Figs 8, 11, 9 and 12), lead to a larger reduction in local soil carbon. However our 50%-100% simulations present the same response as our high and mid latitude results, where local soil carbon increases. This highlights the importance of the initial magnitude of land use change, and resultant climate change, on the soil carbon response to deforestation in the UVic ESCM. The 15%-75% simulations all have converging levels of forest loss by 2100 (Fig 3), thus the rate of change of land cover can also play a role in soil carbon retention. This further enhances the argument that the change in soil carbon arises through a multitude of pathways, which influence the influx and outflux of carbon.

The increase in atmospheric CO_2_, and warmer temperatures seen in our low latitude simulations, are climatic effects more often associated with modelled deforestation. Even then, due mostly to the soil carbon response, CO_2_ and temperature increases are not proportional to deforested area. Seeing as how the location and scale of deforestation can lead to either an increase or decrease to local and global soil carbon, our results call attention to the subtleties of this response and toward a need for a better understanding the complexity of soil carbon dynamics [54].

## 5 Summary and Conclusions

- Global SAT response. High-latitude deforestation leads to cooling proportional to deforested area. Removing trees over the low latitudes causes warming but due to an increase in albedo and increase in the soil carbon pool (see below) this warming is reduced in experiments with large initial deforestation fraction. The SAT response of mid-latitude deforestation is small compared to the other two bands with intermediary deforestation fractions causing warming and larger fractions cooling.
- SAT response over deforested areas. Albedo driven cooling is observed for all latitude bands and all initial deforestation fractions.
- The importance of soil carbon. Atmospheric CO_2_ concentration, and consequently the global temperature response to deforestation, are greatly influenced by post-disturbance changes in soil carbon pools inside and outside deforested areas. It is in large part due to an increase in soil carbon over deforested areas that, irrespective of latitude band, larger initial deforestation scenarios show lower final atmospheric CO_2_ concentrations than intermediate scenarios. Given the large uncertainties associated with the modelled terrestrial carbon cycle [55], our results also point to the need for greater understanding of how organic matter behaves in soils [54] and for the adoption of this new knowledge by terrestrial models.
- Pattern and drivers of soil carbon change. Larger NPP, due mostly to CO_2_ fertilization, is the cause of soil carbon increase over non-deforested areas. In the high-latitude experiments non-deforested areas also gain carbon due to colder and dryer conditions. In the mid- and low-latitude experiments deforested area soils become warmer and dryer, with moisture effects overcoming the opposing temperature impacts and leading to net soil carbon gain, particularly for the higher deforestation fraction scenarios, where local soil temperatures do not increase as much. Among experiments that end up with similar total deforested area, those with larger initial deforestation fractions tend to show larger soil carbon gains and consequently relatively cooler temperatures, an indication that the rate of land cover change is an important determinant of the model’s response to deforestation.
- Drivers of soil temperature change over deforested areas. At high latitudes the tendency is for cooling due to the increase in albedo. In both mid and low latitudes the response is warming caused by a post-deforestation reduction in sensible heat flux.
- Clouds and low-latitude deforestation. The local cooling recorded by our experiments is not seen in simulations of low-latitude deforestation conducted by models that incorporate cloud dynamics. Reliable predictions of the effects of deforestation require understanding the cloud response to land cover change, a problem with yet many uncertainties.
